# Revelation of mechanisms associated with strengthening plant cold tolerance through using exogenous substances

**DOI:** 10.3389/fpls.2025.1478692

**Published:** 2025-04-07

**Authors:** Di Feng, Mingxia Zhang, Jianhua Xu, Qian Gao, Jiao Liu, Caixia Li, Xiaoan Sun, Wanli Xu

**Affiliations:** ^1^ Key Laboratory of Saline–alkali Soil Improvement and Utilization (Saline–alkali Land in Arid and Semiarid Regions), Ministry of Agriculture and Rural Affairs, Institute of Agricultural Resources and Environment, Xinjiang Academy of Agricultural Sciences, Urumchi, China; ^2^ Institute of Plant Protection, Chinese Academy of Agricultural Sciences, Beijing, China; ^3^ Jia Sixie College of Agriculture, Weifang University of Science and Technology, Weifang, Shandong, China; ^4^ Farmland Irrigation Research Institute, Chinese Academy of Agricultural Sciences, Xinxiang, Henan, China

**Keywords:** cold stress, exogenous substances, cold tolerance, chemical regulation, microbial regulation

## Abstract

Cold stress (CS) is one of the main factors that limits the crop or plant growth and development in many regions of the world. Many researchers have been endeavoring to break the natural temperature barrier to grow plants in extremely cold areas or to alleviate erratic cold devastation on crops in temperate or subtropical regions for years. Numerous studies and research papers published recently for the last two decades have proven that exogenous substances (ESs) are effective and practical in helping plants tolerate CS. Here, we systematically summarize and characterize all 72 ESs that have been tried against CS, analyze research hotspots in the Web of Science database from 2000 to 2024 using VOSviewer with the keywords “cold stress” and “exogenous substances”, and grouped them accordingly. Based on their underlying mechanisms, five categories of ESs are clearly defined, described and discussed: 1) enhancement of cell osmotic adjustment, 2) improvement of antioxidant pathways, 3) involvement in phytohormone regulation, 4) promotion of photosynthesis; 5) enrichment of nutritional status. After clarifying these five categories, a detailed plant responses and their possible interactions through a signal cross-talk are proposed and followed by discussions on future perspectives on using ESs to fortify plants against CS. The accumulative knowledge and information provided here will be ultimately used to increase plant productivity and agricultural sustainability under CS through chemical and microbial approaches.

## 1 Introduction

Plants live in an environment with a constantly changing temperature and are greatly affected by its downshift that seriously impacts their normal growth, development and productivity, and limits their geographical habitat (Wang et al., 2017). Approximately, 64% of our earth’s land has the lowest average temperature below 0°C (Rihan et al., 2017), which should include the plants under cold (0 - 15°C) and freezing stress (<0°C). Due to their adaptability through the chronological evolution, plants in tropical and subtropical regions usually do not survive sporadic freezing, while those living in temperate regions are tolerant to cold stress (CS) or resilient against freezing by acclimation (Jeon and Kim, 2013). While being under CS, plants and crops reduce their growth productivity, posing a serious threat to food security (Zhang et al., 2022b). With an intensified global warming and unexpected temperature fluctuations due to climate changes, extreme weather conditions occur more frequently and plants/crops are suffering from increasingly severe colds and freezing calamities, causing a devastating yield loss or prominent crop loss (Liu et al., 2022a). Therefore, it has become of great significance and is imperative to understand how CS undermines plant health and impacts crop production and how plants respond to CS and the underlying mechanisms involved in alleviation of CS through application of exogenous substances (ESs).

With recent advances and achievements in molecular biology and CS researches, the complexity and diversity of plant responses to CS and their corresponding mechanisms have been realized and recognized more clearly for the last two decades, which includes but is not limited to various physiological, biochemical, and molecular changes. First and foremost, plants under CS seem to accumulate excessive reactive oxygen species (ROS) resulting in damage of cell membranes, leakage of cell fluids, impairment of normal physiological functions, increment of malondialdehyde content, peroxidation of plasma membranes, and excessive oxidative activities (Chen et al., 2021). Secondly, the chlorophyll synthesis in plants is hindered, various photosynthetic enzymes are deactivated, photosynthesis is inhibited, and photosynthetic rate decreased (Ding et al., 2019). Thirdly, plants respond to CS with noticeable changes in the content of osmoregulatory substances such as soluble sugars, soluble proteins, and proline as well as the water content of plant cells, resulting in osmotic adjustments (Fàbregas et al., 2020). Fourthly, plants under CS tend to enhance the anaerobic respiration, causing the protein denaturation, abnormal hormone levels, and changes in root activity (Ji et al., 2010) that all impairs water absorption, desiccation of aboveground plant tissues (Aroca and Ruiz Lozano, 2012; Hussain et al., 2018), and consequently reduction of root growth and development (Nezhadahmadi et al., 2013; Hassan et al., 2017). Finally, plants are able to sense coming cold and activate expressions of CS-related genes through signal cross-talks to regulate all responses mentioned above in neutralizing the CS impact (Qari et al., 2022) and alleviating its damages. However, this type of self-regulated responses and tolerance in plants is rather limited and species-dependent.

In order to improve the stress resistance in plants, researchers have made countless attempts in finding promising ESs for that purpose and some of them have proven to be effective and applicable (Feng et al., 2023b). Thus, inducing or enhancing positive responses in plants to reverse CS by ESs has become an important research area aiming at revealing functions of ESs and providing their theoretical underlying mechanisms. Although ESs are not magic cures against plant freezing stress and there is a large amount of research literature on them that needs to be carefully analyzed and summarized, we have mainly focused on analyzing the basic mechanisms of ESs that help plants alleviate CS. So far, 72 ESs have been reported to mitigate plant CS in the literatures published during the last two decades around the world and their relevant mechanisms summarized and detailed (**Table 1**). According to their chemical composition and source (Cheng et al., 2020), ESs are divided into five categories: inorganic salts, organic compounds, plant hormones, plant extracts, and multi-element complexes. While the classification based on chemical composition and source (Cheng et al., 2020) provides one way to categorize ESs, we also adopt another approach considering other aspects. We divide ESs into the chemical or microbial group first and then separate chemical substances into the subgroup of organic (polyamines, polyphenols, peptides, polyols, sugars, esters, hormones, vitamins, amino acids, organic acids, plant growth regulators) and inorganic compounds.

**Table 1:** List of exogenous substances reported in alleviation of cold stress.

Inorganic		
Selenium (Se)	Boron (B)	Phosphorus (P)
Silicon (Si)	Molybdenum (Mo)	Zinc (Zn)
Potassium (K)	Calcium chloride (Cacl_2_)	Carbon dioxide (CO_2_)
Nitric oxide (NO)	Hydrogen peroxide (H_2_O_2_)	Hydrogen Sulfide (H_2_S)
Hydrogen (H_2_)		
Microbes		
*Porkipicus Coccus Bacillus* (PCB)	*Flavobacterium succinicans* (FS)	Arbuscular mycorrhizal fungl (AMF)
*Pseudomonas strain*	*Trichoderma harzianum* (Th)	
Plant growth regulator		
Uniconazole (UNZ)	Paclobutrazol (PBZ)	1-Methylcyclopropen (1-MCP)
Choline chlorid (CC)	Carboxin	Coronatine (COR)
Phthalanilic acid (PA)	Compound Sodlum Nitrophenolak (CSN)	
Hormones		
Melatonin (MT)	Methyl Jasmonate (MeJA)	2,4-Epibrassinolide (EBR)
Ethephon	Abscisic acid (ABA)	Paecilomyces varioti (ZNC)
Strigolactone (SL)	Gibberellins (GAs)	6-benzyladenineng (6-BA)
Cytokinin (CTK)	Jasmonic acid (JA)	
Organic acids		
Arachidonic acid (ARA)	Salicylic acid (SA)	Humicacid
Ferulic acid (FA)	Alginic Acid	Oxalic acid (OA)
Malic acid (MA)		
Amino acids		
γ-Aminobutyric Acid (GABA)	γ-glutamate (γ-PGA)	Glutamic acid
Glycine betaine (GB)	Proline (Pro)	5-Aminolevulinic (ALA)
Saccharides		
Chitosan oligosaccharide (COS)	Chitosan	Sucrose
Trehalose (Tre)		
Esters		
Diethyl aminoethyl (DA-6)	Propyl gallate (PG)	
Polyamines		
Putrescine (Put)	Spermidine (Spd)	Dopamine (DA)
Vitamins		
Zeaxanthin	Vitamin B2, B12 (VB2, VB12)	
Polyols		
Polyethylene glycol (PEG)	Inositol	
Polyphenols		
Gallic acid (GLA)		
Peptides		
Glutathione (GSH)		
Other		
Adenosine triphosphate (ATP)	Pyroligneous acid	Dimethyl Sulfoxide (DMSO)
Ferrotitanium reagent (Tiron)	Phospholipase D (PLD)	Seaweed extract
Moringa leaf extract		

Due to extensive research advances achieved for the last 25 years and accumulative findings available in recent literatures, we have thoroughly reviewed the most recent and outstanding studies that were conducted in using different ESs to alleviate plant CS, summarized their mechanisms involved in responses against different levels of CS through structural, physiological, biochemical, and molecular changes. The review we present here is to shed some light on the future research directions, provide some assistance in finding an appropriate ESs for alleviation of plant CS to some degree, and ultimately solve the problems associated with plants under CS.

## 2 Research development and advance of ESs in alleviating CS in plants

### 2.1 Overview

**Figure 1:**
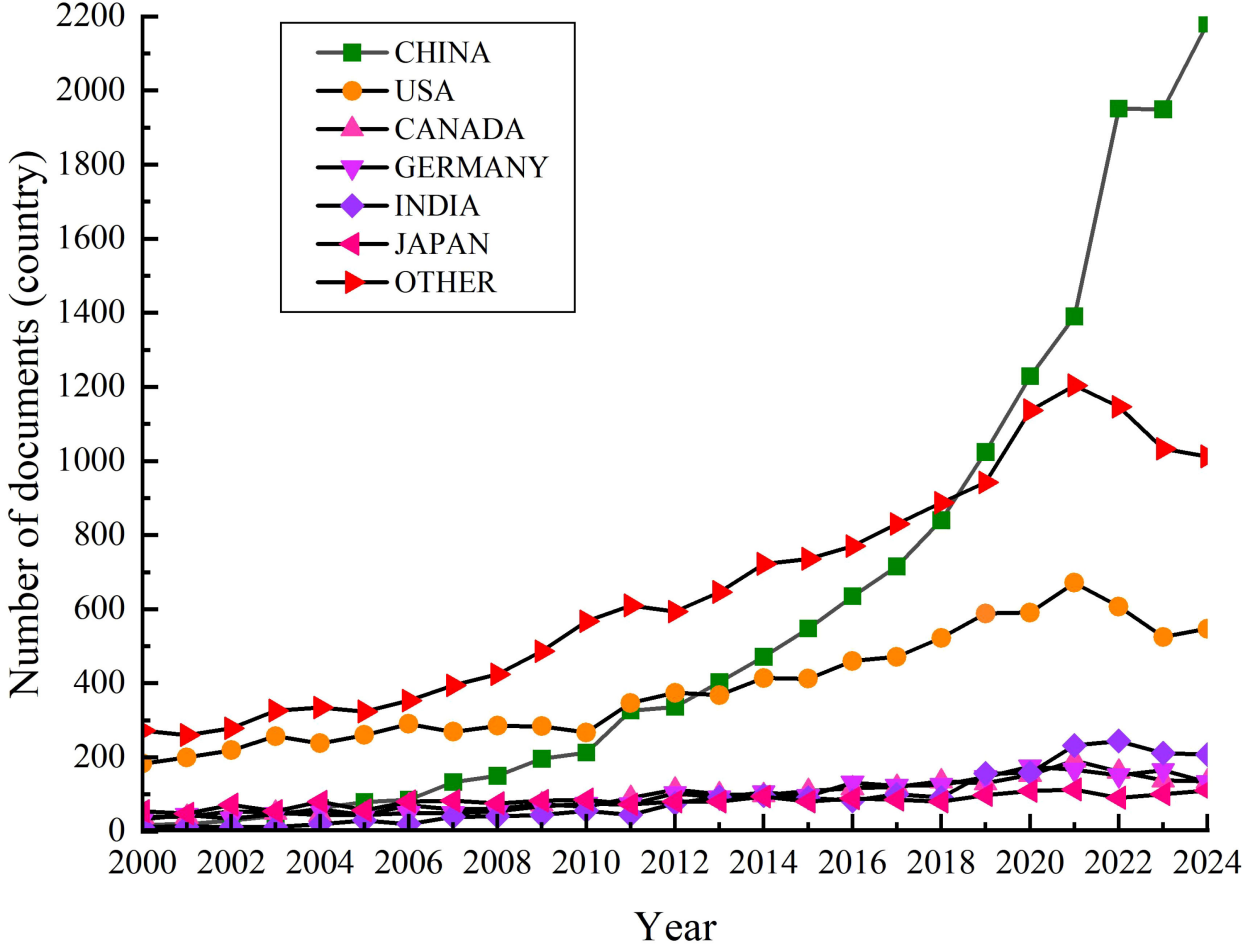
Trends in the number of articles published on cold stress with different countries from 2000 to 2024.

The research on ESs to alleviate plant CS started in the 1940s and has been an international focus in plant biology and physiology. This review uses the keywords “cold stress”, “exogenous substances”, “cold resistance” and “cold tolerance” to search for research papers published between 2000 and 2024 in the Web of Science database. A total of 49956 articles are found and included in the whole collection of the database. From a comparison between countries, Chinese scholars have published a total of 15027 papers (30.1%), American scholars have published 9649 articles (19.3%), Canadian scholars have published 2400 articles (4.8%), German scholars have published 2346 articles (4.7%), Indian scholars have published 2186 articles (4.4%), Japanese scholars have published 2062 articles (4.1%), and the scholars from other countries have published 16286 articles (32.6%). Based on this, a run chart of the number of documents published on cold stress by different countries during 2000 - 2024 has been formed (**Figure 1**). **Figure 1** indicates that more attentions and efforts have been given to the CS studies, such as in China where the number of papers published has increased by more than 100 times in the last 25 years. The global trend of CS research interest also reflects indirectly the frequent occurrence of extreme weather conditions and the increasingly prevalent issues pertaining to the global food availability and demand. So, it has become obvious that the world is paying more attention to plant CS and its possible solution through ESs application.

### 2.2 Analysis of hotspots in international research reports

**Figure 2:**
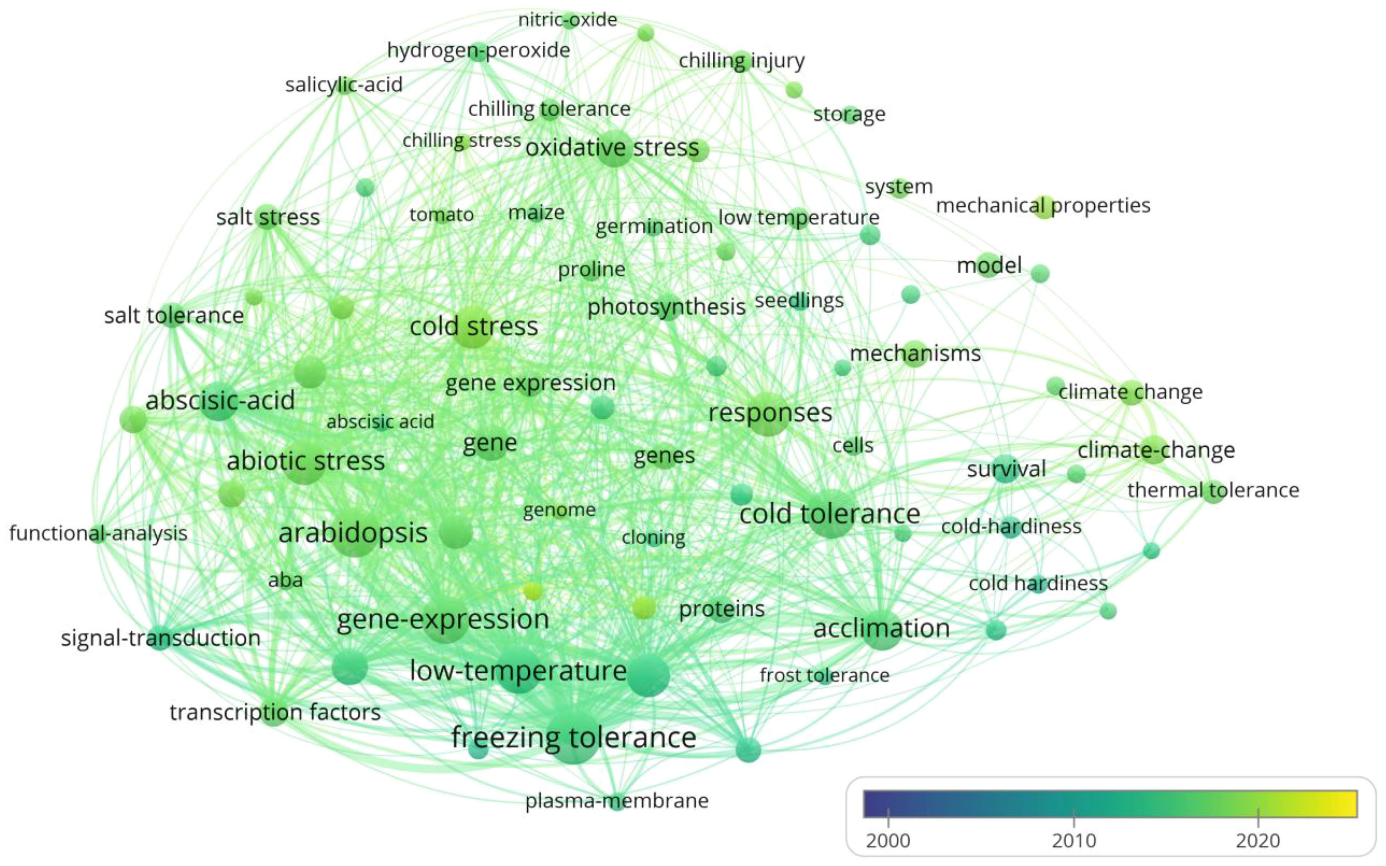
Hotspot analysis of English literatures resulted from searching keywords of “cold stress”, “exogenous substances”, “cold resistance” and “cold tolerance” during last 25 years. The size of each dot represents the focal length of the keywords in the literature, and the line between two points represents closeness of their coupling relationship.

VOSviewer is used to analyze literature collected from the Web of Science database under keywords of “cold stress”, “exogenous substances”, “cold resistance” and “cold tolerance” during last 25 years (2000-2024) for a research hotspot map (**Figure 2**). Six hot topics have emerged from the analysis: crop response, crop varieties, growth stages, climate change, types of ESs, and underlying mechanisms, while Melatonin (MT), Nitric oxide (NO), Abscisic acid (ABA), Salicylic acid (SA), Calcium chloride (CaCl_2_) and ethylene are the most studied ESs to alleviate CS. In terms of the mechanisms associated with the effect of ESs on mitigating CS, “improving antioxidant system and maintaining membrane structure stability”, “hormone regulation”, “enhancing cell osmotic regulation ability”, “improving photosynthetic system” and “improving the nutritional status” are the hot topics derived from the literature analysis. Therefore, we believe that the plant tolerant mechanisms pertaining to effect of ESs on CS alleviation should be separated into 5 groups accordingly: 1) enhancement of cell osmotic adjustment, 2) improvement of antioxidant pathways, 3) involvement in phytohormone regulation, 4) promotion of photosynthesis; 5) enrichment of nutritional status.

## 3 The mechanism of ESs underlying plant CS

### 3.1 Enhancement of cell osmotic adjustment

While being under CS, plants manage to increase their cell fluid, maintain the osmotic pressure in cells, and protect membrane components through accumulating various osmotic adjusting metabolites (Khan et al., 2017) as the first defensive line of cold resistance against an osmotic imbalance likely caused by the exosmosis of cell fluid (Ren et al., 2013). Some ESs have been identified to induce synthesis of osmoregulation substances. For example, spraying propyl gallate (PG) on cucumber plants under CS could increase the soluble sugar content, reduce the cell osmotic potential, and maintain the integrity, structure and function of cucumber proteins in cucumber leaves (Gao et al., 2022). Qian et al. (2021) used 1 μL L^-1^ 1-methylcyclopropene (1-MCP) to fumigate peach fruit for 12 hours, resulting in an increased content of proline (Pro) and phenylpeptide amino acid (PA) for enhanced cold tolerance in fruits through regulating cell osmotic pressure. Also, exogenous proline could be used on pepper callus as a signal molecule to stimulate defense pathways in endogenous proline biosynthesis and improve the accumulation of endogenous proline and antifreeze proteins against CS (Koç, 2013). Spraying 5 mM ferulic acid (FA) on tomatoes under cold stress induced the gene expression of C-repeat binding factors (CBF) in transcription pathway, increased the expression of *SlMAPK3*, *SlCBF1*, and *SlICE1*, and promoted the accumulation of proline and soluble proteins (Shu et al., 2022). Similar effect of ESs on priming chickpea (*Cicer arietinum* L.) seeds for cold tolerance was observed through soaking seeds with 5 µM GAs under CS to maintain a high relative water content and low electrolyte leakage, maintain water balance, and promote the damaged cells to divide and elongate (Aziz and Pekşen, 2020).

Some beneficial microbes have proven to participate in osmotic adjustment through inoculating tomato seeds with *Trichoderma harzianum* AK20G strain to reduce the lipid peroxidation rate and electrolyte leakage and increase the leaf water content and proline accumulation (Ghorbanpour et al., 2018). Arbuscular mycorrhizal fungi (AMF) was also used to colonize plant roots to enhance cold tolerance in rice and to form hyphae vesicles that facilitate rice roots to absorb nutrients, promote proline accumulation through enhancing glutamate (Glu) and ornithine (Orn) synthesis (Liu et al., 2022b).

External application of PLD helps the signaling pathway cope with short-term CS in barley seedlings by regulating the balance between proline and ROS levels. In contrast, the decrease in PLD activity in response to long-term cold stress does not affect proline levels. The lipid signaling triggered by PLD plays a key role in both short-term and long-term cold stress in barley (Peppino Margutti et al., 2017).

So, it can be seen that this CS mitigating mechanism mainly works through improving the cell osmoregulation which is likely related to the antioxidant metabolism and membrane structure. Interestingly, this type of osmotic damages caused by cold may not be as severe as those caused by drought or salt stress, so there are not enough studies on using ESs for osmoregulation. Perhaps that’s why there are relatively fewer studies on using ESs to further enhance osmoregulation substances. However, priming the osmotic regulation ability of seed or seedling cells may be still meaningful for plants to develop a sustainable cold tolerance in the field and needs more attention and effort.

### 3.2 Improvement of antioxidant pathways

Under CS, a large amount of ROS accumulates in plant cells. ROS can act as a signaling substance in cells to induce gene expressions and protein syntheses for cold tolerance in plants (Su et al., 2019), but it can also interfere with normal oxidative activities (Heidarvand and Maali Amiri, 2013), disrupt the stability of proteins or protein complexes, reduce the activity of ROS scavenging enzymes, and lead to photoinhibition of photosynthesis and damaged cell membranes (Siddiqui and Cavicchioli, 2006). Introducing ESs can enhance antioxidant capacity of plants to remove superoxide anions, hydrogen peroxide (H_2_O_2_), malondialdehyde and other peroxides through activating the enzymes involved in the antioxidant pathway and promoting the production of non-enzymatic antioxidant substances (Feng et al., 2023a). Non enzymatic antioxidant substances refer to small molecule compounds with antioxidant properties, including the human metabolite uric acid and intracellular synthesized glutathione, as well as vitamin E, vitamin C, carotenoids (such as astaxanthin, lutein, zeaxanthin, etc.), selenium, copper and other nutrients, and phytochemicals such as tea polyphenols. The effect of ESs on stabilizing the oxidative system does not only help plants avoid potential damages caused by oxidative stress but also effectively protect plant cells from abnormal changes in the membrane spatial configuration, permeability, or leakage. Moreover, some ESs have been found to act as signaling molecules to promote plant cold regulatory and defensive activities against cold (Song et al., 2015). Moreover, some TF families encoding the cold tolerance include *DREB, WRKY, NAC, MYB, AP2, ERF*, and *bHLH* (Yang et al., 2018), among which *CBF1, CBF2, CBF3, CBF4, ICE1, ICE2, CAMTA3, MYB15, ZAT12, COR15a*, and *COR15b* can regulate the cold signaling and cold stress. With the application of ESs, TFs balanced the ROS content in plants through the indirect action, alleviated the oxidative damage of cell membranes, maintained the normal metabolism of plants, and improved cold tolerance (Ritonga and Chen, 2020). Exogenous SA was reported to induce an accumulation or biosynthesis of some extracellular proteins in barley, regulate the activities of cytoplasmic extracellular antioxidant enzymes and ice nucleation, and diversify extracellular proteins for enhanced cold tolerance against CS (Mutlu et al., 2013; Turkyilmaz Unal et al., 2015). Lei et al. (2017) found that γ-Polyglutamic acid (γ-PGA) secreted by bacteria could increase the H_2_O_2_ content in root cells of rapeseed seedlings and transmit H_2_O_2_ to leaves through the Ca^2+^ signaling channel to activate antioxidant enzymes for removal of H_2_O_2_ and various peroxide metabolites in enhancing cold tolerance. The postharvest γ-Aminobutyric acid (GABA) treatment of cucumber fruit was reported to increase antioxidant enzyme activity, reduce ROS accumulation (Malekzadeh et al., 2017), protect cell membranes from impairment due to CS, which is consistent with the findings concluded from the study on exogenous GABA treatment of tomato seedlings under CS (Abd Elbar et al., 2021). Zhang et al. (2021) injected *Flavobacterium succinate* (a plant growth promoting microbe) into the roots of ryegrass seedlings to enhance cold tolerance through the secreted IAA, dissolved phosphorus, and enriched iron carriers to increase the biomass, antioxidant enzyme activity, soluble sugars, and proline content, reduce the relative conductivity and malondialdehyde content, and keep the redox balance in check (Zhang et al., 2021). More examples of the effect of ESs on mitigating CS have shown by using exogenous boron (B) to enhance plant antioxidant enzyme activity and reduce the negative impact of ROS (Ghassemi et al., 2021) and applying exogenous CO_2_ to increase the activity of antioxidant enzymes and the concentration of osmoregulation substances in wheat leaves for an adjustment of cold tolerance in wheat offspring through the sucrose metabolism (Li et al., 2020). It has come to a consensus that the negative effect caused by excessive accumulation of ROS can be alleviated through various ESs applications (**Table 2**), but the relationship between ROS and major metabolites such as proline is still unclear.

**Table 2:** Optimal concentration and application method of exogenous substances used to improve antioxidant system.

Category	Exogenous substance	Optimal concentration and application method	Test plant	Processing temperature	Effect	Reference
Inorganic	Selenium (Se)	2 mg mL^-1^ Foliar spraying	Tea (*Camellia sinensis* L.)	4°C	The malondialdehyde and H_2_O_2_ content decreased by 31.59% and 23.94% respectively. The Pro content decreased by 34.64%. SOD and POD activity increased by 30.41% and 34.44%.	(Liu et al., 2021a)
	Silicon (Si)	0.1/1 mM Seed soaking	Cucumber (*Cucumis sativus* L.)	Day/night temperature at 15/8°C	The increase in endogenous silicon content leads to an increase in antioxidant activity such as SOD, GSH-Px, APX, MDHAR, GR, GSH, and AsA.	(Liu et al., 2009)
	Molybdenum (Mo)	1% Seed soaking	Wheat (*Triticum aestivum* L.)	4°C	A significant increase in the SOD, CAT, and GPX activity.	(Al Issawi et al., 2016)
	NO	1 mM Sodium Nitroprusside (SNP) Foliar spraying	Cucumber (*Cucumis sativus* L.)	4°C	Both electrical conductivity (EC) and malondialdehyde decreased, while Pro content, CAT, SOD, and GR activities increased.	(Liu et al., 2011)
	H_2_O_2_	10 mM Seed soaking	Tangerines (*Citrus reticulata* Blanco)	4°C	In 12 hrs, the CAT activity increased by 175.38% and APX activity increased 156.52%.	(Afrin et al., 2018)
	H_2_S	0.5 mM NaHS Foliar spraying	Blueberries (*Vaccinium* spp.)	4~6°C	The Pro content increased by 32.69% and the malondialdehyde content decreased by 19.15%.	(Tang et al., 2020)
Plant growth regulator	Uniconazole (UNZ)	50 mg L^-1^ Foliar spraying	Urad (*Vigna radiata* L. Wilczek)	15°C	In 4 days, the accumulation of EL, malondialdehyde, O^2−^ and H_2_O_2_ decreased by 279%, 989%, 262% and 243% respectively. The activity of SOD, POD, APX and GR increased by 0.71-10.00%, 1.35-29.26%, 4.47-25.33% and 2.48-21.21%, respectively.	(Yu et al., 2022)
	Choline chlorid (CC)	500 mg L^-1^ Foliar spraying	Wheat (*Triticum aestivum* L.)	0°C	The activity of SOD, POD and CAT increased by 28.92%, 41.03%, 25.56%, and the Pro content increased by 44.62%.	(Jing et al., 2018)
	Coronatine (COR)	0.01 µM Seed soaking	Paddy (*Oryza sativa* L.)	4°C	Regulating the antioxidant defense system and reducing the toxic effect of reactive oxygen species on cells.	(Feng et al., 2022)
	Phthalanilic acid (PA)	100 mg L^-1^ Foliar spraying	Maize (*Zea mays* L.)	5°C	The malondialdehyde content decreased to 14.34%-51.50%, and the electrolyte leakage rate decreased to 13.30%-35.46%.	(Yang et al., 2022)
Hormones	Melatonin (MT)	100 µM Seed soaking	Eggplant (*Solanum melongena* L.)	13°C	After 3 days of treatment, the malondialdehyde content decreased by 34.11%, while the soluble protein, SOD, POD, CAT, and APX content increased by 45.46%, 406.98%, 356.78%, 204.08%, and 84.52%, respectively.	(Cao et al., 2019)
	*Paecilomyces varioti* extract (ZNC)	20 ng ml^-1^ Foliar spraying	Cabbage (*Brassica campestris* L.)	4°C	SOD, POD, and CAT activities increased by 29.41%, 69.37%, 153.48%, H_2_O_2_ content decreased by 23.69%, malondialdehyde concentration decreased by 28.07%, and Pro concentration increased by 6.25%.	(Wang et al., 2022a)
	Gibberellins (GAs)	1 mg L^-1^ Invitro culture	Black fritillary (*Fritillaria* spp.)	4°C	The activity of SOD and CAT increased by 281% and 1400%, respectively.	(Petrić et al., 2013)
	6-benzyladenineng (6-BA)	20 mg L^-1^ Foliar spraying	Paddy (*Oryza sativa* L.)	16°C	The activity of POD and SOD increased.	(Wang et al., 2022b)
Organic acids	Arachidonic acid (ARA)	2.5 mg L^-1^ Fruit soaking	Banana (*Musa nana* Lour.)	0°C	The malondialdehyde content and EL level decreased.	(Wan et al., 2022)
	Salicylic acid (SA)	100 µM Foliar spraying	Wheat (*Triticum aestivum* L.)	4°C	SOD activity and CAT activity increased by 90% and 15%, respectively.	(Ignatenko et al., 2019)
	Humic acid	0.05% Seed soaking	Cucurbita pepo (*Cucurbita pepo* L.)	5°C	The activity of SOD and POD increased by 22.1% and 48.2%, respectively. The soluble sugar and proline content increased by 54% and 50.2%, respectively.	(Li et al., 2023)
	Oxalate (OA)	5 mM Seed soaking	Apricot (*Prunus armeniaca* L.)	2 ± 1°C	The H_2_O_2_ and malondialdehyde content decreased, while the soluble sugar content increased.	(Wang et al., 2016)
	Malic acid (MA)	80 mM Seed soaking	Banana (*Musa nana* Lour.)	6°C	Increase the activity of peroxidase (POD) and polyphenol oxidase (PPO) to enhance the scavenging activity of free radicals.	(Huang et al., 2016)
Amino acids	Glutamic acid	2.5 mM Foliar spraying	Tomato (*Solanum lycopersicum* L.)	Day/night temperature at12/9°C	Increasing the content and/or activity of antioxidant enzymes, reducing oxidative damage, and increasing the expression level of genes encoding antioxidant enzymes and C-receptor binding factors (CBFs).	(Lee et al., 2021)
	Glycine betaine (GB)	30 mM Foliar spraying	Tomato (*Solanum lycopersicum* L.)	4°C	Tomato seedlings treated with GB removed excess ROS, reduced the accumulation of malondialdehyde, peroxides, and superoxide anions, and increased the activity of SOD, POD, and CAT.	(Dai et al., 2024)
	5-Aminolevulinic (ALA)	0.6 mM Foliar spraying	Soybean (*Glycine max* L. Merr.)	10 ± 0.5°C	The activity of SOD and CAT increased and Pro content increased.	(Manafi et al., 2015)
Saccharides	Chitosan oligosaccharide (COS)	100 mg L^-1^ Foliar spraying	Peanut (*Arachis hypogaea* L.)	8°C	The malondialdehyde and H_2_O_2_ content decreased by 47.27% and 60.30% respectively with an 121% increase in the SOD and CAT activity. The activity of SOD and CAT increased by 67.25% and 44.01%, respectively.	(Shi et al., 2023)
	Sucrose	6% Hydroponics	Strawberry y (*Fragaria ananassa* Duch.)	4°C	The seedling survival rate increasd by 50%.	(Lukoševičiūtė et al., 2009)
	Trehalose (Tre)	10 mM Foliar spraying	Muskmelon (*Cucumis melo* L.)	Day/night temperature at 15/6°C	The activity of H_2_O_2_ APX and GR and NO level increased. The REC content decreased by 11.7%.	(Liu et al., 2021b)
Polyamines	Dopamine (DA)	100 μM Root drench	Watermelon (*Citrullus lanatus* (Thunb.) Matsum. & Nakai)	Day/night temperature at 10/5°C	In 8 days, the Pro content increased by 58.28%, the malondialdehyde content decreased by 26.50%, and the activity of SOD, POD and CAT increased by 35.13%, 119.60% and 132.91%, respectively.	(Jiao et al., 2021)
Vitamins	Vitamin B2, B12 (VB2 VB12)	100 mg Seed soaking	Maize (*Zea mays* L.)	5°C	In 7 days, The SOD activity increased by 3.62% and the POD activity increased by 40.31%.	(Xin Chi et al., 2021)
Polyols	Polyethylene glycol (PEG)	30% Foliar spraying	Maize (*Zea mays* L.)	8°C	Malondialdehyde content decreased by 37.93%.	(Pu et al., 2015)
Polyphenols	Gallic acid (GLA)	1 mM Foliar spraying	Soybean (*Glycine max* L. Merr.)	5°C	The CAT activity increased and the APX activity increased by 48%.	(Yildiztugay et al., 2017)
Peptides	Glutathione (GSH)	1 mM Foliar spraying	Tomato (*Solanumlycopersicum* L.)	Day/night temperature at 10/3°C	The content of H_2_O_2_ decreased by 330%. The CAT activity was enhanced by 1830%, the glutathione reductase (GR) content increased by 440%, SOD increased by 214%, and AXP increased by 280%.	(Gul et al., 2022)
other	Adenosine triphosphate (ATP)	25 μM Foliar spraying	Rape (*Brassica napus* L.)	4°C	The activity of SOD, POD, CAT, and APX antioxidant enzymes increased by 14.1%, 31.3%, 44.3%, and 39.7%, respectively. The content of Pro and soluble sugar increased by 40.2% and 45.9%, respectively.	(Hu et al., 2021)
	Dimethyl Sulfoxide (DMSO)	20% PEG, 0.5% DMSO, 0.007% SA Foliar spraying	Maize (*Zea mays* L.)	8°C	The POD and CAT content increased by 42.7% and 118.0%, respectively.	(Sun et al., 2014)
	Ferrotitanium reagent (Tiron)	10 mM Foliar spraying	Autumn eggplant (*Kandelia candel* L. Druce)	5°C	SOD, POD, and CAT activities increased by 130%, 130%, and 120%, respectively.	(Pan et al., 2022)

### 3.3 Phytohormone regulation

Plant hormones are essential in regulating plant growth, development and reproduction serving also as signaling molecules that respond to stress and widely participating in crop physiological processes as cross-talk messengers (Li et al., 2017). It has been noticed that plant cold resistance is induced and primed through a disruption of the hormone balance to begin with (Huang et al., 2021) and any ES that interfere with the phytohormone balance has the potential to be used for adjustment of plant cold tolerance. Under CS, plant growth hormones (such as auxin, gibberellin, cytokinin, etc.) usually sense changes such as reduced metabolism, obstructed transportation and disturbed signal transduction, leading to an inhibition of plant growth. However, low temperature always reduces the water availability in plant cells, causing osmotic stress and stimulating the ABA synthesis (Raza et al., 2023). ABA proves to play an important role in many cellular processes. Endogenous ABA is transported from roots to leaves and accumulates in guard cells to regulate stomatal closure and reduce the transpiration rate and cell growth (Raza et al., 2023). A foliar application of exogenous ABA helps activate the antioxidant defense system mediated by nitric oxide (NO), prevent excessive accumulation of ROS, and alleviate oxidative damage caused by cold stress (Dong et al., 2017). Therefore, some ESs used to adjust the phytohormone balance in plants under CS can improve plant cold tolerance. Exogenous MT was recognized to activate the endogenous hormone metabolic pathways, promote the amount of endogenous hormones SA, JA, Auxin (IAA), and reduce the levels of ABA, ethylene (ETH), and GAs, which is conducive to the growth and development of red bean seedlings under CS (Chen et al., 2021). Exogenous Methyl Jasmonate (MeJA) proves to promote the expression of genes that modulate the jasmonic acid biosynthesis in plants under CS, leading to an increase in endogenous jasmonic acid content, thereby enhancing the expression of three jasmonic acid responsive transcription factors and improving artemisinin biosynthesis (Liu et al., 2017). Zuo et al. (2022) used 15 mM Pro to soak corn seeds and found that Pro alleviated the decrease in IAA, zeatin nucleoside, and GA content in corn embryos, reduced the increase in the ABA level, which alleviated the inhibitory effect of cold on seed germination. The application of Zinc enhanced the synthesis of cytokinin (CTK) and IAA in rice tillering buds under CS, promoted the transportation of IAA from tillering buds to other parts of rice plants, and improved the resistance of rice against CS (Liu et al., 2022d). Exogenous 2, 4 epibrassinolide (EBR) was found to increase the content of GAs and IAA in tomato seedlings, accelerated the tomato growth rate, and alleviated the damage caused by CS (Heidari et al., 2021). Spermidine (Spd) promoted wheat seed germination by regulating the content of abscisic acid and gibberellin, as well as starch degradation (Gu et al., 2019).

**Figure 3:**
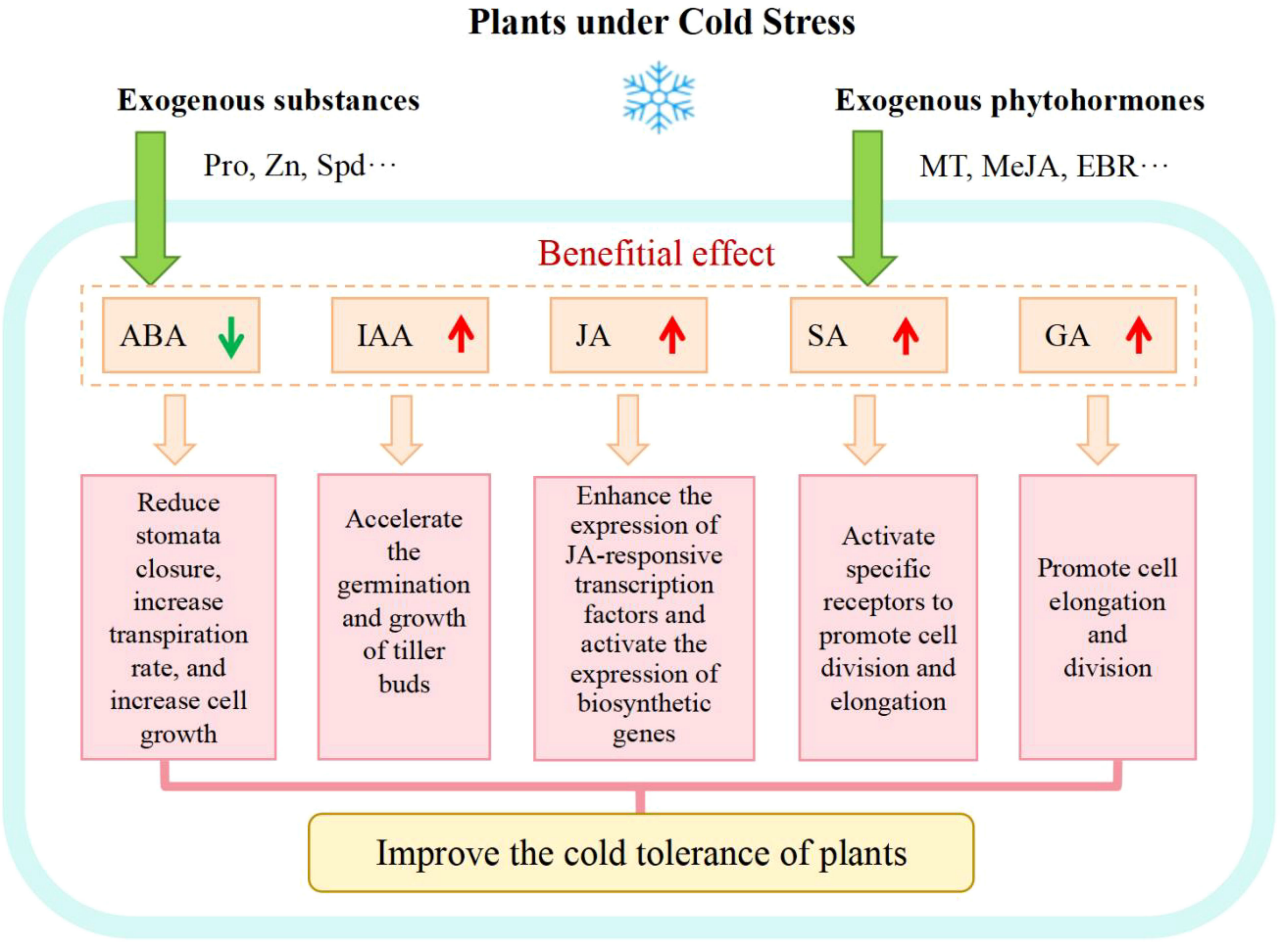
Mechanisms of exogenous substances involved in enhancing plant cold tolerance.

Mechanisms of exogenous substances involved in enhancing plant cold tolerance are shown in **Figure 3**. At present, research on cold tolerance through regulating endogenous hormone levels in plants through ESs should have drawn more attention and investment and the interactive pathways between endogenous and exogenous hormones should be further explored.

### 3.4 Promotion of photosynthesis

As a key physiological process in plants, photosynthesis is highly sensitive to and regulated by temperature fluctuations (Zhu et al., 2018) due to the involvement of many catalytic enzymes. Cold impacts on the photosynthetic system including the photosynthetic pigments, photosynthetic parameters and chlorophyll fluorescence responses, likely affecting all components involved such as the structure and function of chloroplast, stomata, chlorophylls, transpiration rate (Tr), intercellular CO_2_ concentration, apparent quantum efficiency, dark respiration rate, photochemical efficiency, non-photochemical quenching coefficient, chlorophyll fluorescence parameters enzyme activity, photosynthetic electron transfer, carbon assimilation process, and photochemical reactions (Li and Cui, 2018; Lambers et al., 2008). Researches have proven that suitable ESs are useful to alleviate CS impacts on the photosynthetic system. Applying SA during the winter wheat seedling stage significantly improved the photosynthesis and photochemical efficiency of the photosystem II, effectively alleviated CS impacts on the net photosynthetic rate, plant height, biomass, and yield, and significantly prolonged the wheat growth window (Wang et al., 2021a). Exogenous Tre also proved to be effective on salvage impairments of wheat grain due to CS at booting stage. The alleviation obviously occurred through enhancing nitrogen assimilation, increasing the content of endogenous Spd, ascorbic acid (GSH) and AsA, promoting the GSH/AsA cycle, and alleviating an inhibition of wheat floret development (Liang et al., 2021). Moreover, additional exogenous phosphorus (P) fertilizer helped slow down the senescence of wheat flag leaves, increase green leaf area and photosynthetic capacity, improve assimilation accumulation, promote the transportation of assimilates to grains, thereby reduce wheat yield loss due to CS (Xu et al., 2022). Overall, the chlorophyll content is the basis of photosynthesis and reflects the photosynthetic efficiency of a plant, therefore, there is a need for systematic researches on such chlorophylls as indicators of photosynthetic effects regulated by ESs, and their corresponding changes of photosynthetic characteristics. The ESs related to improving the photosynthetic system and their optimal application concentrations are detailed in **Table 3**.

**Table 3:** The optimal concentration of exogenous substances for partial improvement of photosynthesis.

Category	Exogenous substance	Optimal concentration and application method	Test plant	Processing temperature	Effect	Reference
Inorganic	Selenium (Se)	5 mg L−1 Foliar spraying	Strawberries (*Fragaria × ananassa* Duch.)	0°C	Pn increased by 102.6% and Chl increased by 34.81%.	(Huang et al., 2018)
	Calcium chloride (CaCl_2_)	15 mM Foliar spraying	Peanuts (*Arachis hypogaea* L.)	8°C	Significantly increase transpiration rate (Tr) and net photosynthesis rate (Pn), stomatal conductance(Gs), and intercellular CO_2_ concentration (Ci).	(Liu et al., 2013)
	Hydrogen Sulfide (H_2_S)	0.5 mM NaHS (H2S donor) Foliar spraying	Blueberries (*Vaccinium* spp.)	4~6°C	The chlorophyll concentration, stomatal aperture, and density increased to enhance the photosynthetic capacity.	(Tang et al., 2020)
	Hydrogen (H_2_)	0.39 mM Foliar spraying	Rice (*Oryza sativa* L.)	0°C	Increasing of the chlorophyll a, chlorophyll b, and total chlorophyll content and enhancement of Pn and Gs.	(Xu et al., 2017)
Plant growth regulator	Uniconazole (UNZ)	50 mg L^−1^ Foliar spraying	mung beans (Vigna radiata L.)	15°C	AsA, DHA, GSH, GSSG, total ASA and total GSH increased by 12.78-30.78%, 5.61-33.16%, 9.35-25.91%,3.44-6.24%, 2.24-37.43%, 3.22-19.05%, respectively.	(Yu et al., 2022)
	Paclobutrazol (PBZ)	75 mg L−1 Foliar spraying or Root drench	Pomegranates (*Punica granatum* L.)	4 ± 1°C	The carbohydrate content increased by 35.98%.	(Moradi et al., 2017)
	Carboxin	7.2% Lagging cover	Cotton (*Gossypium* spp.)	12°C	The net photosynthetic rate, stomatal conductance, and intercellular CO_2_ concentration increased by 11.0%, 17.0%, and 9.0%, respectively.	(Xiao yun et al., 2020)
	Compound Sodlum Nitrophenolak (CSN)	50 mg L−1, 100 mg L−1 Seed soaking, Root drench	Cucumbers (*Cucumis sativus* L.)	Day/night temperature at 12/6°C	The content of chlorophyll a, chlorophyll b, total chlorophyll, and carotenoid increased by 25.2%, 23.5%, 24.3%, and 8.9%, respectively, with a significant increase of 25% in Pn and 12% in Ci.	(Huang et al., 2022)
Hormones	Melatonin (MT)	100 μM Foliar spraying	Tomatoes (*Solanum lycopersicum L.*)	Day/night temperature at 15/6°C	Increasing of the Fv/Fm ratio and maximum photochemical efficiency of PSII.	(Yang et al., 2018)
	2,4-Epibrassinolide (EBR)	0.1 μM Foliar spraying	Cheese (*Solanum melongena* L.)	Day/night temperature at 10/5°C	The concentration of ASA and GSH increased by 13.7% and 22.0%, respectively.	(Wu et al., 2015)
	Ethephon	1 mg L-1 Foliar spraying	Sugar cane (*Saccharum officinarum* L.)	5°C	The sucrose content decreased by 31.14%; the ATPase activity increased by about 3-3.4 times, and the activity of reducing sugar, acid invertase, ATPase, IAAO, and NR increased.	(Rai et al., 2008)
	Abscisic acid (ABA)	150 μM Seed soaking	Cucumbers (*Cucumis sativus* L.)	Day/night temperature at 15/8°C	The content of stachyose, raffinose, sucrose, fructose, and glucose increased by 1740%, 560%, 560%, 230%, and 270%, indicating an increase in the STS activity.	(Meng et al., 2008)
	Strigolactone (SL)	2 μM Seed soaking	Arabidopsis(*Arabidopsis thaliana* L. Heynh.)	4°C	Significant increase in the chlorophyll a/b ratio and carotenoid content, as well as an increase in the photosynthesis rate.	(Cooper et al., 2018)
Organic acids	Alginic acid	0.1 g L-1 Foliar spraying	Tobacco (*Nicotiana tabacum* L.)	4°C	Maintaining of a high photosynthesis and the normal pigment content in leaves.	(Shi et al., 2013)
Amino acids	5-Aminolevulinic (ALA)	25 ppm Foliar spraying, Seed soaking	Chili (*Capsicum annuum* L.)	3°C	The content of Chl, sucrose, RWC, Gs increased.	(Korkmaz et al., 2010)
Esters	Diethyl aminoethyl (DA-6)	10 mg L-1 Hydroponics	Corn (*Zea mays* L.)	9~15°C	On the 3rd, 5th, and 7th day, the total chlorophyll content increased by 4.91%, 9.92%, 9.66%, respectively, while Tr decreased by 23.48%, 26.03%, 32.06%.	(Zhang et al., 2020)
Vitamins	Zeaxanthin	50 mg L-1 Foliar spraying	Chili (*Capsicum annuum* L.)	Day/night temperature at 15/5°C	Ci increased by 37.84%.	(Ding et al., 2022)
Polyols	Inositol	5 mM Root drench	Periwinkle (*Catharanthus roseus* L. *G.* Don)	4°C	The content of chlorophyll a, total chlorophyll, and Car increased by 50.69%, 44.31%, and 69.67%, respectively.	(Wei et al., 2021)
Other	Pyroligneous acid	0.25% Foliar spraying	Rape (*Brassica napus* L.)	Day/night temperature at 10/5°C	Stomatal density and water use efficiency increased by 14% and 55%, respectively, while intercellular the CO_2_ concentration and stomatal conductivity decreased by 9% and 41%, respectively.	(Zhu et al., 2022)
	Seaweed extract	4% Foliar spraying	Tea (*Camellia sinensis* L.)	5°C	The thickness of leaves, palisade tissue, and sponge tissue increased by 63.84%, 18.72%, and 8.86%, respectively. After 40 days of spraying, the chlorophyll content in tea leaves increased by 97.88%.	(Yu et al., 2021)
	Spicy wood leaf extract	3% Foliar spraying	Moringa (*Moringa oleifera* Lam.)	12.9~20.1°C	After two foliar applications, there was a significant increase in the number of branches (92%), leaves (141%), leaf like leaves (61%), chlorophyll a (51%) and b (61%), total chlorophyll (54%), membrane stability index (60%), and leaf phenolic content (63%).	(Batool et al., 2019)

### 3.5 Enrichment of nutritional status

Under CS conditions, plant growths and metabolisms are significantly affected due to a reduced or limited absorption and utilization of minerals and nutrients. In plants, coldness hinders physiological activities and slows down all metabolisms, especially in roots. It also decreases the permeability of cell membranes throughout the whole plants (Zhang and Sonnewald, 2017). To help plants fight against CS, some ESs have demonstrated their property in enriching the nutritional status in plants so as to alleviate CS. They can be used as nutrients themselves alone or as to promote the nutrient absorption and transport, redistribution and metabolic activities. In that regard, some beneficial microbes have been found to help plants grow and develop normally under CS such as plant growth promoting bacteria (PGPB) that were considered to stimulate nitrogen fixation, nutrient dissolution, phytohormone synthesis and to regulate iron carrier production, chitinase activity, antibiotic and cyanide production in plants (Selvakumar et al., 2010). Supplementing Put in plants under CS activated the arginine polyamine pathway in red palm, induced the synthesis of endogenous arginine (Arg), improved the synthesis and conversion rate of endogenous arginine, and reduced the decomposition rate of endogenous arginine (Sun et al., 2021). Rai et al. (2008) found that the application of potassium (K), ethephon and zinc (Zn) to sugarcane at 5°C increased the germination rate by 80%, 50% and 40%, respectively, promoted the activities of sugar - reducing enzymes, acid invertase, ATPase, indole acetic acid oxidase (IAAO) and nitrite reductase (NR), and enhanced the oxidative irritability and nitrogen content to stimulate the growth of buds. Pepper seeds treated with chitosan demonstrated an enhanced activity of chitinase and glucanase that thereby increased the germination rate and better protected seedlings from fungal diseases under CS (Samarah et al., 2020). As a beneficial bacterium in soil, *Bacillus subtilis* was able to colonize, grow and help absorb nutrients through participating in the nitrogen fixation, secretion of antibiotics, activation of proteases, and dissolution of inorganic and organic phosphorus in roots of maize seedlings (Zhang et al., 2022a). Similarly, *Pseudomonas* strains that were introduced to roots proved to synthesize IAA in vegetables under CS by stimulating the seed and tuber germination, triggering the plant cell division and proliferation, and controlling the vegetative growth to alleviate CS damages (Tsavkelova et al., 2006; Spaepen and Vanderleyden, 2011). Overall, most studies on enrichment of nutritional status with ESs have focused on the absorption and utilization of nutrients, and further researches should be aimed to improve nutrient redistribution so that more nutrients to be available and utilized for growth and development for plant cold tolerance.

## 4 Summary

**Figure 4:**
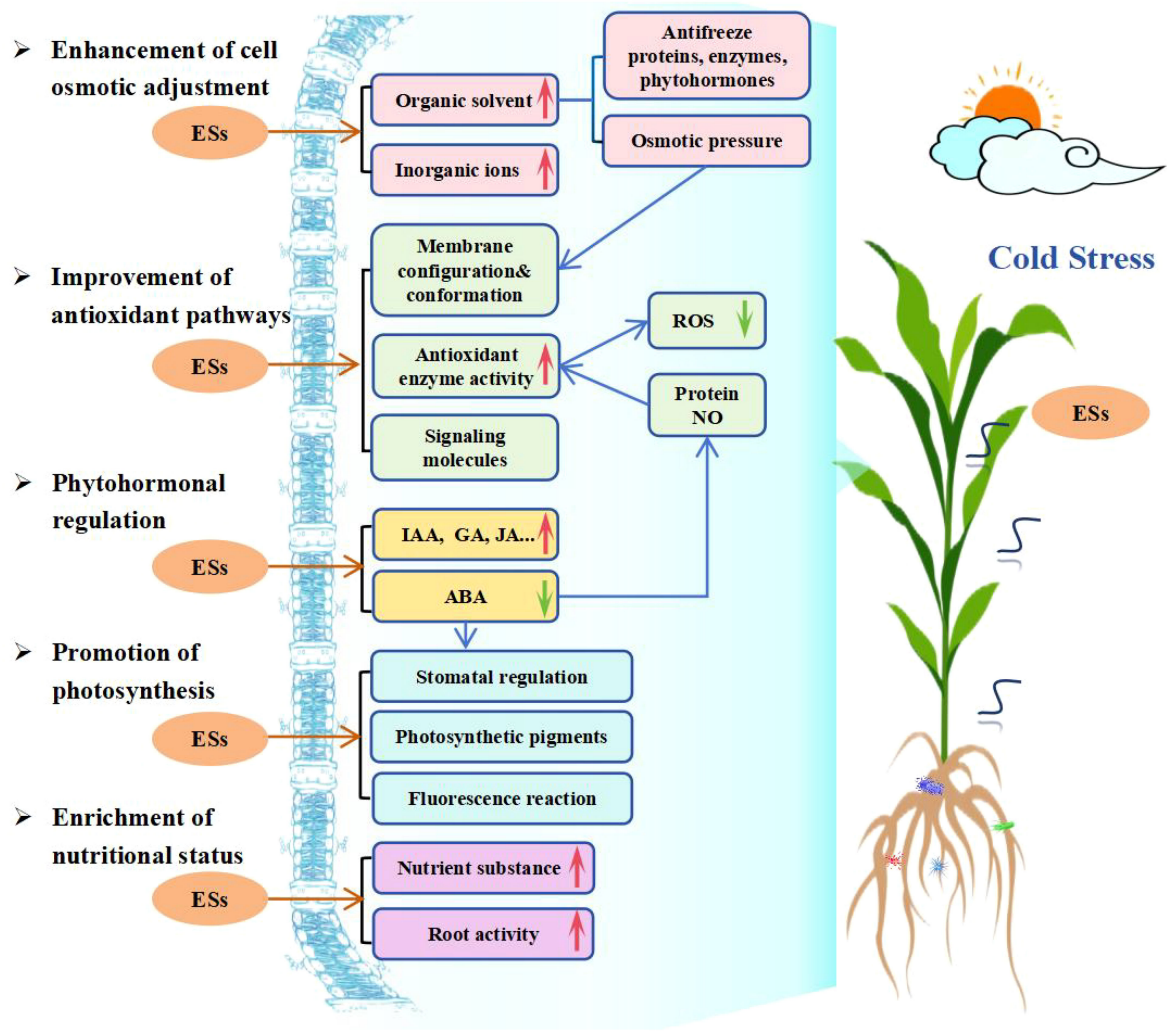
Mechanisms of exogenous substances (ESs) involved in enhancing plant cold tolerance. Red and green arrows indicate promotion/increase or inhibition/decrease, respectively.

With a global climate change and frequent occurrence of extreme weather, the plant CS is inevitable. Research has shown that ESs play a positive role in enhancing plant tolerance/resistance against CS and therefore has drawn many researchers’ attention to determine on mechanisms involved in both plant cold tolerance against CS and application of ESs in alleviating adversary effects of CS. Up to date, about 72 ESs have been identified and evaluated for their possible use in plants to tolerate CS during the past 25 years and ESs research efforts have expanded from a few chemicals for structural, morphological and preliminary physiological adaptations at the beginning to the current status involving in various ESs on biochemical, molecular, and microbial functions and their interactions and crosstalks through signal transmission throughout the entire regulatory system. This review of over a hundred research papers here has grouped mechanisms of ESs in alleviating plant CS into 5 categories with a summarized elaboration (**Figure 4**). However, due to the differences and uniqueness of each plant species, the whole picture of a plant’s adaptation to cold through using ESs to adjust its morphological traits, internal physiological structures, gene regulation and biochemistry is not fully drawn, therefore, the undertakings of the researches on ESs will continue, especially when extreme temperature downshifts become more frequent.

## 5 Outlook

For ESs to achieve their expected efficacy in CS alleviation, the application method can be critically important. So far, the foliar spraying, seed soaking, root irrigation, hydroponics, and inoculation are five application methods that have been used. Among those, leaf spraying seems to be the most commonly used application, especially on seedlings due to the fact that leaves provide the largest area to receive sprayed ESs and can respond to them instantly. However, ESs should be applied according to their chemical properties, the action sites of ESs, type of crops, and growth stage of plants since the function, mechanism and efficacy of ESs may vary under certain circumstances. At present, there is no unified standard for the amount and duration of use of ESs in most studies, resulting in a lack of reference basis for the use of ESs and a bias in understanding the effects of ESs. So, this review has provided a detailed list of the optimal application method, concentration, plant growth stage that are derived from literatures and can be used as a reference provided any of those ESs are intended to be used for further studies. In addition, in case there are situations where multiple stresses occur simultaneously, use of couple or multiple ESs can be attempted to induce more resilience in plants against abiotic stresses, and for that purpose, this review can be used to find relevant information of each individual ESs. In addition, most studies on using ESs to alleviate CS in plants are conducted on seedlings in a laboratory or greenhouse settings and a field trial should be more informative and practical to verify the efficacy or “priming” effect of ESs on field crops.

Cold stress drives plants to respond with the structural and physiological adjustments through sensing the temperature downshift, transmitting a stress signal to interior of cells, and ultimately inducing gene expressions in response to CS. Certain ESs demonstrate their efficacy in inducing the expressions of certain genes encoding for cold tolerance and antifreeze proteins including their precursors (Rayirath et al., 2009). However, all these plant responses to CS relating to gene expressions are presumably initiated by the cold receptors that sense the low temperature and turn on the “molecular switch”. CBF seems to be one of those protein receptors activated under CS and subsequently to trigger a series of signal responsive pathways and interactions among various defensive mechanisms, activate the expression of multiple downstream CS responsive genes, enhance cold resistance, and enable plants to adapt and survive under CS. Therefore, the CBF signaling pathway can be a reinforcement of plant tolerance against CS other than the preexisted mechanisms such as structural adaptation, adjustment of membrane lipids, production of antifreeze proteins, etc. Moreover, any ES that affects gene expressions, such as CBF should be explored further to induce a possible and temporary “dormancy” in plant seedlings prior to the incoming low temperature with reduced metabolisms and a minimized growth rate for their tolerance against temporary CS.

This review has elaborated in great detail on positive effects of ESs on alleviation of plant CS, but we strongly believe that they are not sufficient and effective to mitigate the damages brought upon by extreme cold/freezing conditions beyond plant can endure. Finally, we suggested that some research areas be further focused and endeavored to reveal more fundamental responses and their underlying mechanism in plants against CS: 1) the relationship between ROS and major metabolites such as proline; 2) the interaction and constraint pathways between endogenous hormones that are regulated by ESs; 3) the changes pertaining to photosynthetic processes that are regulated by ESs; 4) the post transcriptional process and post translational modification of plant cold tolerance genes; and 5) the low-temperature cold signal regulatory network and cold signal transduction/crosstalks in other crops.

Overall, this review theoretically summarizes the underlying mechanisms of ESs involved in enhancing cold tolerance and alleviating CS in plants and provides comprehensive perspectives for the future research with paramount knowledge and various directions on chemistry, microbiology, plant physiology, and molecular biology. With information collectively provided in the review, further research can be focused on developing actual work plans and novel ES-based strategies to CS related issues and possible impact on plant growth, crop production, yield loss and food security.
